# Exhaled Breath and Oxygenator Sweep Gas Propionaldehyde in Acute Respiratory Distress Syndrome

**DOI:** 10.3390/molecules26010145

**Published:** 2020-12-31

**Authors:** Agnes S. Meidert, Alexander Choukèr, Siegfried Praun, Gustav Schelling, Michael E. Dolch

**Affiliations:** 1 Department of Anesthesiology, University Hospital of Munich—Campus Großhadern, Ludwig-Maximilians-University of Munich, 81366 Munich, Germany; agnes_meidert@web.de (A.S.M.); alexander.chouker@med.uni-muenchen.de (A.C.); gustav.schelling@med.uni-muenchen.de (G.S.); 2 V&F Analyse-und Messtechnik GmbH, Andreas Hofer Strasse 15, 6067 Absam, Austria; siegfried.praun@vandf.com; 3 Department of Anesthesiology & Intensive Care Medicine, InnKlinikum Altötting, Vinzenz-von-Paul-Str. 10, 84503 Altötting, Germany

**Keywords:** acute respiratory distress syndrome, extracorporeal membrane oxygenation, oxidative stress, propionaldehyde, breath gas analysis

## Abstract

Background: Oxidative stress-induced lipid peroxidation (LPO) due to neutrophil-derived reactive oxygen species plays a key role in the early stage of the acute respiratory distress syndrome (ARDS). Monitoring of oxidative stress in this patient population is of great interest, and, ideally, this can be done noninvasively. Recently, propionaldehyde, a volatile chemical compound (VOC) released during LPO, was identified in the breath of lung transplant recipients as a marker of oxidative stress. The aim of the present study was to identify if markers of oxidative stress appear in the oxygenator outflow gas of patients with severe ARDS treated with veno-venous extracorporeal membrane oxygenation (ECMO). Methods: The present study included patients with severe ARDS treated with veno-venous ECMO. Concentrations of acetone, isoprene, and propionaldehyde were measured in inspiratory air, exhaled breath, and oxygenator inflow and outflow gas at corresponding time points. Ion-molecule reaction mass spectrometry was used to measure VOCs in a sequential order within the first 24 h and on day three after ECMO initiation. Results: Nine patients (5 female, 4 male; age = 42.1 ± 12.2 year) with ARDS and already established ECMO therapy (pre-ECMO PaO_2_/FiO_2_ = 44.0 ± 11.5 mmHg) were included into analysis. VOCs appeared in comparable amounts in breath and oxygenator outflow gas (acetone: 838 (422–7632) vs. 1114 (501–4916) ppbv; isoprene: 53.7 (19.5–244) vs. 48.7 (37.9–108) ppbv; propionaldehyde: 53.7 (32.1–82.2) vs. 42.9 (24.8–122) ppbv). Concentrations of acetone, isoprene, and propionaldehyde in breath and oxygenator outflow gas showed a parallel course with time. Conclusions: Acetone, isoprene, and propionaldehyde appear in breath and oxygenator outflow gas in comparable amounts. This allows for the measurement of these VOCs in a critically ill patient population via the ECMO oxygenator outflow gas without the need of ventilator circuit manipulation.

## 1. Introduction

Acute respiratory distress syndrome (ARDS) is characterized by diffuse alveolar damage, alveolar capillary leakage, and a protein-rich alveolar exudate in response to an excessive inflammatory process. This presents clinically as ventilation-perfusion mismatch with severe hypoxemia, bilateral infiltrates, and decreased pulmonary compliance [1].

ARDS might either develop from direct lung injury, such as pneumonia and aspiration, or indirect lung injury, such as sepsis or trauma with shock and transfusion. Independent from direct or indirect lung injury, an overwhelming inflammatory process leads to alveolar epithelial and endothelial injury and finally results in alveolo-capillary barrier dysfunction and failure [2]. Neutrophil-derived oxidants play a key role in the pathogenesis of ARDS, as reactive oxygen species (ROS) rapidly react with polyunsaturated fatty acids located in cell membranes [3]. In this way, a lipid peroxidation (LPO) termed process is initiated that triggers a chain reaction resulting in cellular dysfunction or even apoptosis. During this process, several aldehydes and alkanes are released as degrading products [4]. Due to their physiochemical properties, these compounds are volatile (VOCs) and appear in the breath, where they can be measured noninvasively. Considering the key role of ROS and LPO in the development and progress of ARDS, monitoring of volatile markers of LPO represents an interesting alternative to standard markers of inflammation.

Among these compounds, pentane has been identified recently as a possible marker of oxidative stress in patients at risk of and with ARDS by gas chromatography mass spectrometry [5]. In addition to this, propionaldehyde was detected in response to severe, ischemia-reperfusion-induced oxidative stress in a group of lung transplant recipients using ion-molecule reaction mass spectrometry (IMR-MS) [6]. Despite the noninvasive character of breath gas analysis, access to the patient’s breath via the endotracheal tube is required. As a consequence of airway manipulation, accidental loss of positive end expiratory pressure might occur, which poses the risk of lung collapse and hypoxemia [7,8]. In addition, the disconnection of the endotracheal tube leads to exposure of healthcare workers with potentially infectious aerosols, e.g., in patients suffering from highly infectious diseases such as COVID-19 pneumonia. Although this can be avoided by careful handling, alternative sites for the measurement of exhaled breath VOC content are desirable. Furthermore, the predominantly dependent location of pulmonary infiltrations and collapsed lung areas in ARDS patients results in a significant ventilation–perfusion mismatch with shunt perfusion. Consequently, the elimination of VOCs via the lungs might be impaired and exhaled breath VOC content might not exactly reflect concentrations found in blood.

The hypothesis of the present study was that propionaldehyde, a marker of oxidative stress recently detected in exhaled breath, can be measured in the oxygenator outflow gas of ECMO systems. Therefore, patients with ARDS requiring ECMO support were included in this pilot study. In addition, acetone and isoprene were analyzed.

## 2. Results

### 2.1. Study Population

Ten ARDS patients with the need for ECMO support were included in this pilot study. Measurements in one patient could not be used for technical reasons. All patients had pneumonia as the common cause of severe ARDS. Patients 6 and 7 were ventilated for interstitial lung disease and developed pneumonia progressing to ARDS. None of the patients underwent surgery or received volatile anesthetics in the previous 4 days before measurements. Survival to hospital discharge was 66.7%, and one-year survival was 55.6% with one patient dying due to arterial tracheostoma bleeding after transfer to a rehabilitation clinic. Table 1 and Table 2 show the baseline characteristics and respiratory parameters at the time of the first measurement.

### 2.2. IMR-MS Measurements

Measurements of breath and oxygenator gas were performed in all patients within the first 24 h after initiation of ECMO support. Follow-up measurements were possible in eight patients on day 3 after ECMO initiation. No adverse events occurred during breath and oxygenator gas measurements.

**Figure 1:**
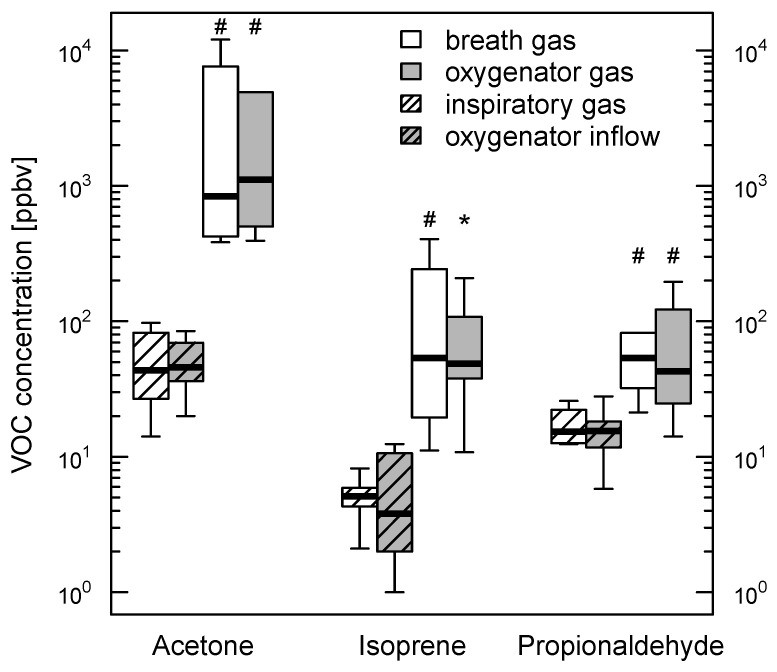
Breath and oxygenator gas VOC content. Concentrations of acetone, isoprene, and propionaldehyde in breath and extracorporeal membrane oxygenator gas on day one of measurements. Note that the concentrations found in oxygenator outflow gas are comparable to those found in exhaled breath (VOC, volatile organic compound). Intragroup comparisons with Wilcoxon signed-rank test. # *p* < 0.005; * *p* < 0.01.

**Figure 2:**
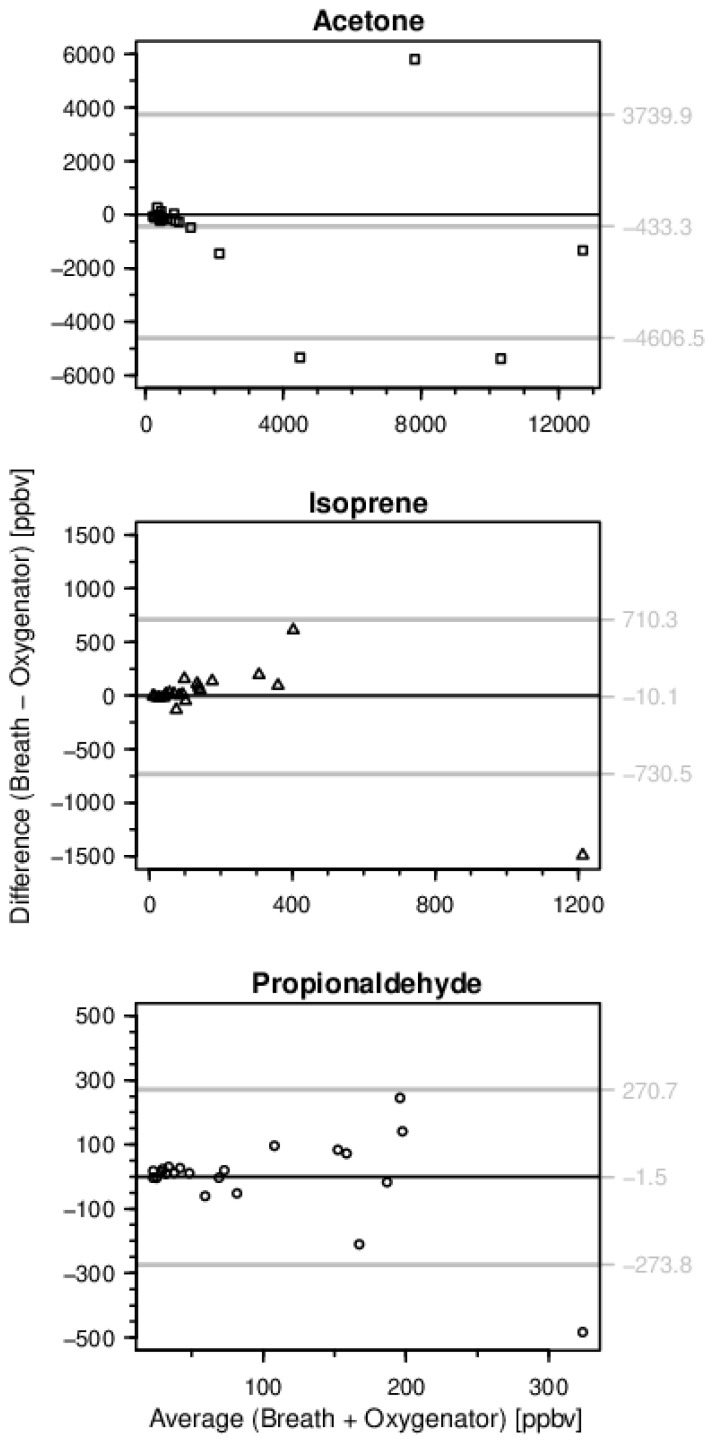
Bland-Altman plot of exhaled breath and oxygenator outflow gas VOC content. Concentrations of acetone, isoprene, and propionaldehyde in breath and extracorporeal membrane oxygenator gas on day one and three of measurements are included. For moderate VOC concentrations a generally good agreement of VOC concentrations in breath and oxygenator gas was observable. The grey lines represent the limits of agreement (average difference ± 1.96 standard deviation of the difference).

**Figure 3:**
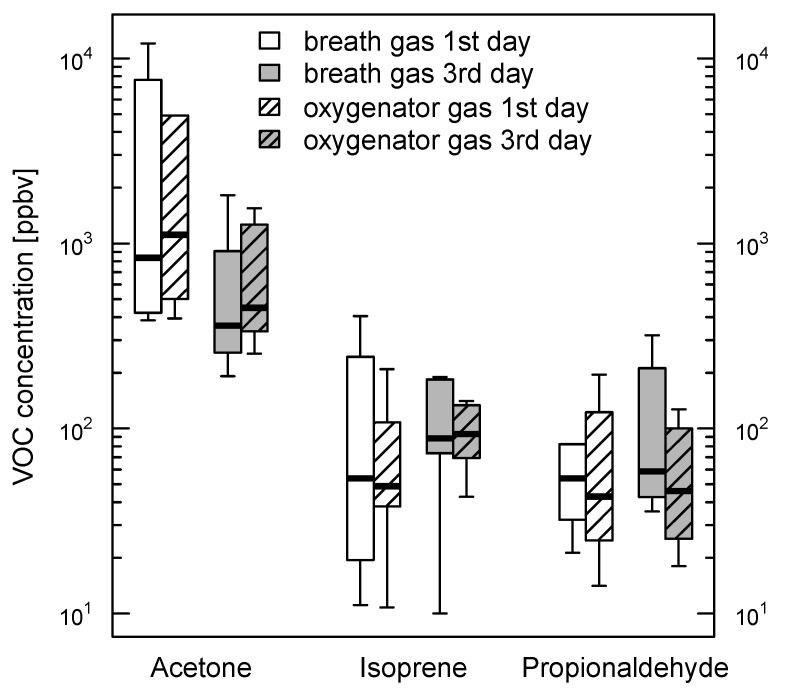
Time-dependent decrease in exhaled VOCs. Temporal evolvement of acetone, isoprene, and propionaldehyde in exhaled breath and oxygenator outflow gas. Note that volatile organic compounds (VOCs) measured in oxygenator outflow gas course are nearly parallel to the values in exhaled breath.

Concentrations of acetone, isoprene, and propionaldehyde are shown in Figure 1. All VOCs analyzed appeared in exhaled breath as well as in oxygenator outflow gas in comparable amounts. This also applies for the measurement results obtained in inspiratory respectively oxygenator inflow gas. The best agreement of VOC concentrations measured in breath gas and oxygenator outflow gas was obtained for moderate VOC concentrations. For higher concentrations, there was a trend towards increased differences predominately observed in acetone and propionaldehyde measurements (Figure 2). The temporal change of exhaled breath and oxygenator outflow gas VOC concentrations is shown in Figure 3. Propionaldehyde, acetone and isoprene concentrations in exhaled breath and oxygenator outflow gas changed in parallel on days one and three. In patients 6 and 7 with pre-existing interstitial lung disease, exhaled breath and oxygenator outflow gas propionaldehyde showed marked differences when compared with those of the patients without preexisting lung disease (breath propionaldehyde 38.6 (31.0–46.1) vs. 67.3 (40.6–130.2) ppbv; oxygenator propionaldehyde 35.1 (31.2–39.0) vs. 70.8 (21.7–158.9) ppbv; median with 25th–75th percentiles) without reaching statistical significance.

### 2.3. Inflammation and Lung Function

Plasma interleukin-6 values remained unchanged when comparing day one and day three (346.0 (48.0–5992.0) vs. 130.2 (39.45–916.0) pg/mL; *p* = 0.15). In addition, no significant changes were observed when comparing measurements on day one and three of lung injury score (4.0 (3.8–4.0) vs. 3.5 (3.4–3.8); *p* = 0.10), pulmonary dynamic compliance (17.5 (15.2–20.0) vs. 23.4 (21.2–30.8) mL/mbar; *p* = 0.46), and ECMO blood flow (3.1 (3.0–3.8) vs. 2.9 (2.7–3.2) L/min; *p* = 0.40); all values as median with 25th–75th percentiles; Wilcoxon signed-rank test.

## 3. Discussion

The main finding of the present study is that propionaldehyde, a marker of oxidative stress, along with acetone and isoprene, is detectable in oxygenator outflow gas of ARDS patients depending on veno-venous ECMO support. Furthermore, the concentrations of propionaldehyde, acetone, and isoprene in oxygenator outflow gas were comparable to those in exhaled breath. This finding possibly allows access to patient’s “breath gas” at the ECMO oxygenator without the need for ventilator circuit manipulation.

Oxidative stress-induced LPO has been shown previously to contribute to cell death and disease progress in patients suffering from ARDS, critical illness, and solid organ transplantation [2,3,9]. Recently, several markers of LPO were identified in breath. Breath ethane was identified as a specific marker of ischemia–reperfusion-induced LPO in the breath of pigs with liver ischemia and in patients with orthotopic liver transplantation [10,11]. In addition to these findings, Scholpp et al. showed that in patients with ARDS or at risk of ARDS, the concentrations of breath pentane and blood malondialdehyde are increased [5]. Following the data on the impact of LPO on propionaldehyde generation in response to experimental myocardial injury and ultraviolet radiation-induced skin LPO, our group recently detected propionaldehyde in lung transplant recipients and critically ill patients exposed to significant ischemia–reperfusion injury and inflammation [6,12,13]. The concentration of propionaldehyde found in our ARDS patients’ breath corresponds well with our previous results obtained in critically ill patients and are well below the concentrations present in lung transplant recipients, a patient population with an extreme form of ischemia–reperfusion injury [6]. In addition, the concentrations of acetone and isoprene found in our ARDS population correspond well with previously reported values in critically ill patients [6]. It is of note that the concentrations of propionaldehyde in patients with pre-existing interstitial lung disease were markedly although not significant reduced when compared with patients without pre-existing lung disease.

As expected, all VOCs investigated appeared in breath as well as in oxygenator outflow gas. Most interestingly, the concentrations of propionaldehyde, acetone, and isoprene appeared in comparable amounts, which possibly allows the utilization of oxygenator outflow gas as an additional or alternative site for “breath gas” analysis. This important observation is well explained by the fact that veno-venous ECMO is by principle connected in series with lung perfusion. Blood containing VOCs originating from the lungs, the intestine, and lower body perfusion returns via the vena cava inferior, where venous drainage for ECMO support takes place. As a result, about 50% of the patient’s cardiac output is drained and passes the ECMO oxygenator where gas exchange takes place. Although a generally good agreement for VOC measurements in breath and oxygenator outflow was found, this close relationship weakened with increasing VOC concentrations. A possible explanation for this finding might be the presence of ventilation–perfusion mismatches leading to decreased pulmonary elimination of VOCs or a decrease in oxygenator functionality. In contrast to veno-venous ECMO, different results might be observed when comparing measurements in breath and oxygenator outflow gas of patients treated with veno-arterial ECMO. During veno-arterial ECMO, lung perfusion is dramatically reduced, and nearly the whole cardiac output passes through the ECMO oxygenator. However, this is of hypothetic nature as we did not perform measurements in patients with veno-arterial ECMO support.

Our findings are partially in accordance with the work of Leopold et al. [14], who investigated VOCs from the external outlet of the ECMO and the outlet of the ventilator’s tube. In contrast to our study, they used a qualitative approach aiming to detect any VOC rather than quantifying specific molecules of interest. In their study, the signal of acetone and isoprene detected in both measurement sites correlated well, whereas propionaldehyde was not identified as a significant signal. Ongoing research focuses on alternative detection methods for VOCs in patients’ breath that are more cost effective and portable than the IMR-MS technology [15,16].

A probable source of concerns regarding the measurement of VOCs over artificial membranes might be the appearance of solvents in oxygenator outflow gas and expiratory breath gas. Recently, Lee et al. investigated the impact of hemodialysis (HD) on expiratory breath VOCs content [17]. In summary, the concentrations of 10 hydrocarbons and 4 halocarbons rapidly increased within 3 min after the onset of the HD procedure. A detailed search identified the polyvinyl chloride (PVC) tubing and dialyzers as the major source of these VOCs. In our patient population, no measurements were performed on ECMO tubing and oxygenator membranes. However, the detailed investigation of Lee and colleagues did not identify propionaldehyde, acetone and isoprene as outgassing products of PVC tubing and dialyzers [17]. Furthermore, they noted a rapid decrease in VOCs originating from PVC tubing and dialyzers within 3 h. This finding makes it very unlikely that propionaldehyde, acetone, and isoprene in oxygenator outflow gas of ECMO treated patients are due to PVC tubing and oxygenator membrane outgassing, as VOC concentrations after ECMO initiation and on day three were comparable. Furthermore, no differences in exhaled breath propionaldehyde were notable when comparing patients without and with the need for ECMO support after lung transplantation [6]. In addition to this, the concentrations of propionaldehyde found in ECMO-treated patients correspond well with values obtained in a general critically ill patient population without the need for ECMO support [6].

Our study has some limitations. First, we examined oxygenator outflow gas and expiratory breath gas on the first and third day of ECMO treatment; therefore, we cannot conclude on the long-term reliability of the approach. Second, the number of patients is small, which may affect the validity of the statistical tests and the generalization of our results.

## 4. Materials and Methods

### 4.1. Ethical Approval and Informed Consent

The institutional review board of the Medical Faculty of the University of Munich approved the study (IRB No: 089/04). In all cases, written informed consent was given by a legal representative.

### 4.2. Patient Population and Treatment

This pilot study included ARDS patients requiring veno-venous ECMO support to overcome acute live threatening hypoxemia or hypercapnia. Exclusion criteria were refusal of the patients’ legal representative and preceding anesthesia with volatile anesthetics.

Diagnosis of ARDS followed the Berlin definition [18]. At our institution, all ARDS patients are treated according to a standardized treatment protocol. This includes adequate sedation and analgesia, antibiotic therapy if indicated by microbiological findings or suspected infection, lung protective mechanical ventilation, prone positioning when indicated on chest computed tomography, and maintenance of adequate perfusion pressure and cardiac output. If life threatening hypoxemia or severe respiratory acidosis persists or develops, veno-venous ECMO support is initiated.

The drainage cannula (Maquet HLS Cannulae, Maquet Cardiopulmonary AG, Hirrlingen, Germany) is usually inserted into the inferior vena cava via the femoral vein, and the return cannula is placed into the right internal jugular vein. The ECMO system consists of a polymethylpentene oxygenator (PLS-QuadroxD; Maquet Cardiopulmonary AG) with an integrated heat exchanger and a total gas exchange surface of 1.8 m^2^, driven by a Rotaflow Centrifugal Pump (Maquet Cardiopulmonary AG). The system is heparin coated, making pronounced systemic anticoagulation unnecessary. Patients received heparin before and during ECMO therapy, monitored by activated partial thromboplastin time (range: 40–50 s).

### 4.3. Ion-Molecule Reaction Mass Spectrometry

A detailed description of the IMR-MS (AirSense Compact, V&F Analyse- und Messtechnik GmbH, Absam, Austria) has been published previously [19]. In brief, a primary ion beam generated by electron-impact ionization of either krypton, xenon, or mercury is used for ionization of sample gas molecules. Selection of primary ions depends on the required ionization energy for sample molecule ionization. Within an octopole system, the primary ion beam induces ionization of sample gas molecules by charge transfer. Thereafter, a quadrupole filter is used for mass-to-charge ratio (*m*/*z*) separation of sample gas molecules. Detection of ions is performed by a downstream located channel electron multiplier system. For the present study, only Hg^+^ with an ionization potential of 10.44 eV was used. The IMR-MS operates under high vacuum (10^−5^ mbar) conditions, and sample gas flow to the ionization unit is controlled to allow for a constant flow. Mass separation is of 1 u over the mass range, the cycle time is 2 ms on average, and the response time is less than 100 ms. The concentration drift in the signal (measured against a steady test gas concentration) is below 5% over a period of 12 h for acetone [19]. Sample gas is transferred in a 2.5 m heated capillary system (Silcosteel, Restek, Bellefonte, PA, USA) at a flow rate of 50 mL/min to the instrument.

### 4.4. Molecules of Interest and Calibration

Acetone, isoprene, and propionaldehyde were measured with a dwell time of 500 ms in a sequential order. Prior to measurements, calibration of the instrument was performed using a standardized calibration gas-containing acetone at 1010 ppbv, isoprene at 1000 ppbv, and propionaldehyde at 12,000 ppbv in balance gas nitrogen 5.0. For background calibration, nitrogen 5.0 was used. A detailed description of the measurement of propionaldehyde has been published previously [6].

### 4.5. Breath Gas Measurements

Measurements in breath were performed over a period of 30 min in exhaled breath and 10 min in inspiratory gas. For the measurement of expiratory gas, a sterilized stainless-steel T-piece connected to the heated transfer line of the IMR-MS was inserted next to the patients’ endotracheal tube. Similarly, a second T-piece was inserted into the inspiratory limb of the ventilator circuitry for measurements in inspiratory gas. A CO_2_-controlled measurement mode integrated into the IMR-MS was used to identify the alveolar part of the breath [19]. The use of disinfectants was abandoned during the last 60 min prior to measurements.

### 4.6. Oxygenator Gas Measurements

For measurement of oxygenator inflow and outflow gas, an 18 gauge Teflon cannula was attached to the tip of the IMR-MS transfer line and inserted into the oxygenator fresh gas supply line and gas outflow orifice. In general, pure O_2_ was used as oxygenator inflow gas. The measurement setup was the same as for breath gas measurements with the exception of a reduced measurement time of 10 min each due to the absence of breath-cycle-associated changes in gas composition.

### 4.7. Statistical Analysis

All data were tested for normal distribution using the Shapiro–Wilk test. Parametric data were reported as mean ± standard deviation, nonparametric data as median with 25–75% percentiles. Statistical significance of differences was tested with the Kruskal–Wallis rank sum test followed by the post hoc Nemenyi test if indicated. Intragroup changes were tested by paired *t*-test or Wilcoxon signed-rank test where appropriate. A *p* value < 0.05 was considered significant. Statistical analyses were carried out with R version 2.11.1 [20].

## 5. Conclusions

In summary, this pilot study showed that propionaldehyde, a marker of oxidative stress and LPO, along with acetone and isoprene, appears in ECMO oxygenator outflow gas. The concentrations of VOCs present in oxygenator outflow gas were comparable with those in expiratory breath gas. This allows ”breath gas analysis” to be performed in this critically ill patient population at a different sampling site without the need of airway circulatory manipulation, which may pose a potential risk for healthcare workers and patients, respectively. Furthermore, measurements in oxygenator outflow gas are not limited by the presence of ventilation–perfusion imbalances, as it is common for pulmonary ventilation–perfusion conditions to exist in patients with severe lung disease like ARDS. Further investigations will have to clarify the impact of changes in cardiac output–ECMO blood flow ratio as well as oxygenator gas exchange functionality on VOC elimination.

## Figures and Tables

**Table 1:** Characteristics of patients at the time of first breath gas analysis.

No	Age (y)	Gender (f/m)	BMI (kg·m^−2^)	CRP (mg·mL^−1^)	Leukocytes (G·mL^−1^)	NE (µg·kg^−1^·min^−1^)	RRT	SAPS II	Outcome
1	38	m	29.2	345	12.0	0.28	-	21	1
2	37	m	27.7	221	2.6	0.14	1	29	1
3	53	m	26.3	19.0	13.1	0.42	-	38	1
4	55	m	26.3	280	1.8	1.11	-	46	0
5	42	f	62.5	153	10.8	0.08	-	33	1
6	36	f	22.0	172	8.9	0.06	-	17	1
7	54	f	22.0	95.0	10.0	0.28	-	41	0
8	56	f	52.0	197	7.4	0.33	-	42	1
9	17	f	25.7	85.0	1.5	0.05	1	43	0

BMI, body mass index; CRP, C-reactive protein; NE, norepinephrine; RRT, renal replacement therapy; SAPS II, simplified acute physiology score.

**Table 2:** Respiratory parameters at the time of first breath gas analysis.

No	V (days)	PaO_2_·FiO_2_^−1^ (mmHg) *	PEEP (mbar)	PIP (mbar)	TV (mL)	TV-IBW (mL·kg^−1^)	MV (L·min^−1^)	ECMO	
								BF (L·min^−1^)	SG (L·min^−1^)
1	1	40	16	36	400	5.2	3.4	4.2	6.0
2	0.25	30	18	26	450	5.6	3.5	3.0	5.0
3	0.6	53	16	30	180	2.4	1.3	2.8	8.0
4	0.4	45	20	34	475	6.2	3.1	4.2	7.0
5	3	70	20	36	280	5.5	2.8	3.1	7.0
6	56	40	17	31	140	2.5	0.7	2.6	3.0
7	16	41	17	33	250	4.5	4.2	3.8	5.0
8	1	37	20	39	320	5.3	4.1	3.7	5.0
9	8	40	20	35	267	4.3	1.6	3.0	6.0

V, duration of mechanical ventilation prior to extracorporeal membrane oxygenation; PaO_2_, partial pressure of oxygen; FiO_2_, fraction of inspired oxygen; PEEP, positive end-expiratory pressure; PIP, peek inspiratory pressure; TV, tidal volume; TV-IBW; TV ideal body weight; MV, minute ventilation; ECMO, extracorporeal membrane oxygenation; BF, ECMO blood flow; SG, ECMO sweep gas flow; * PaO_2_·FiO_2_^−1^ at the time of decision for ECMO initiation.

## Data Availability

The data presented in this study are available on request from the corresponding author. The data are not publicly available due to ethical reasons.

## Sample Availability

Samples of the compounds are not available from the authors.
